# National Cancer Database report of nonmetastatic esophageal small cell carcinoma

**DOI:** 10.1002/cam4.1712

**Published:** 2018-11-06

**Authors:** Vivek Verma, Richard L. Sleightholm, Penny Fang, Jeffrey M. Ryckman, Chi Lin

**Affiliations:** ^1^ Department of Radiation Oncology Allegheny General Hospital Pittsburgh PA; ^2^ Department of Radiation Oncology University of Nebraska Medical Center Omaha NE; ^3^ Department of Radiation Oncology University of Texas M.D. Anderson Cancer Center Houston TX

**Keywords:** chemotherapy, esophageal cancer, radiation therapy, small cell carcinoma, surgery

## Abstract

**Background:**

Esophageal small cell carcinoma (ESCC) is a rare malignancy for which there is no consensus management approach. This is the largest known analysis of nonmetastatic ESCC patients to date, evaluating national practice patterns and outcomes of surgical‐based therapy vs chemoradiotherapy (CRT) vs chemotherapy alone.

**Methods:**

The National Cancer Data Base was queried for esophageal cancer patients with histologically confirmed nonmetastatic ESCC. Univariable and multivariable logistic regression ascertained factors associated with receipt of surgical‐based management. Kaplan‐Meier analysis evaluated overall survival (OS) and the log‐rank test is used to compare OS between groups; Cox univariate and multivariate analyses determined variables associated with OS.

**Results:**

Altogether, 323 patients were analyzed; 64 (20%) patients underwent surgical‐based therapy, 211 (65%) CRT, and 48 (15%) chemotherapy alone. On multivariable analysis, no single factor significantly predicted for administration of surgery. Despite no OS differences between the surgery‐based (median OS 21 months) and CRT arms (18 months), both were superior to CT alone (10 months) (*P* < 0.001). Among other factors, receiving any local therapy independently predicted for higher OS over chemotherapy alone on Cox multivariate analysis (*P* < 0.001).

**Conclusions:**

This study of a large, contemporary national database demonstrates that most ESCC is treated with CRT in the United States; adding local therapy to systemic therapy may be beneficial to these patients, although individualized multidisciplinary management is still recommended.

## 1 INTRODUCTION

Esophageal small cell carcinoma (ESCC) is a rare but highly aggressive malignancy that accounts for less than 2% of esophageal neoplasms.^1^, ^2^ Due to its rarity, there are many accepted treatment paradigms. One approach is to treat as esophageal squamous cell or adenocarcinoma, with definitive chemoradiotherapy (CRT), CRT followed by surgery, or surgery alone.^3^ Another is to utilize nonsurgical treatment (eg chemotherapy (CT) with or without radiotherapy (RT)) similar to that used for small cell carcinoma of the lung. National guidelines have not delineated the optimal treatment strategy for this malignancy.^3^

The somewhat conflicting literature, largely consisting of lower‐volume reports, may also contribute to the lack of consensus. One study of 64 patients (26 nonmetastatic cases) at Memorial Sloan‐Kettering Cancer Center suggested that combined surgery/CT was most associated with reducing recurrences,^4^ with other data (121 nonmetastatic cases) suggesting the addition of RT increases survival.^5^ Both of the aforementioned studies inherently have notable biases, such as the increased proportion of metastatic disease in the former and the lack of chemotherapy records in the latter. A literature review of 199 patients (93 nonmetastatic cases) demonstrated that addition of CT to local therapy (surgery and/or RT) improved outcomes, with no difference in efficacy between either surgery or RT when used as local therapy.^6^ However, a major limitation of that investigation was the lack of comparing local therapy/CT with chemotherapy alone. Lastly, another larger study of 126 patients (85 nonmetastatic cases) demonstrated similar survival with surgery/CT (with or without RT) as compared to CRT; although this was numerically higher than CT alone (no statistical comparisons), the study lumped nonmetastatic and metastatic cases together, and nearly all patients with metastatic disease received CT alone.^7^

Hence, owing to the rarity of this neoplasm, there are virtually no higher‐volume studies specifically evaluating management of nonmetastatic ESCC. As such, national databases may be of high utility to evaluate practice patterns and outcomes. This investigation of purely nonmetastatic ESCC patients, the largest report to date, utilized the contemporary National Cancer Data Base (NCDB), which is estimated to capture 70% of the United States cancer population.^8^

## 2 MATERIALS AND METHODS

The NCDB is a joint project of the Commission on Cancer (CoC) of the American College of Surgeons and the American Cancer Society, which consists of de‐identified information regarding tumor characteristics, patient demographics, and patient survival for approximately 70% of the US population.^8^, ^9^, ^10^, ^11^, ^12^, ^13^, ^14^, ^15^, ^16^, ^17^, ^18^, ^19^, ^20^, ^21^, ^22^, ^23^, ^24^, ^25^, ^26^, ^27^, ^28^ All pertinent cases are reported regularly from CoC‐accredited centers and compiled into a unified dataset, which is then validated. The NCDB contains information not included in the Surveillance, Epidemiology, and End Results database, including details regarding use of systemic therapy. The data used in the study were derived from a de‐identified NCDB file (2004‐2014). The American College of Surgeons and the CoC have not verified and are neither responsible for the analytic or statistical methodology employed nor the conclusions drawn from these data by the investigators. As all patient information in the NCDB database is de‐identified, this study was exempt from institutional review board evaluation.

Inclusion criteria for this study were patients with newly‐diagnosed, histologically‐confirmed and nonmetastatic ESCC. Histologic criteria referred to the International Classification of Disease for Oncology codes of 8041‐8045 or 8073 (representing small cell or oat cell carcinoma). All patients were clinically without metastasis (M0). Exclusion criteria were unknown M classifications or those patients receiving no therapy or palliative treatments. Patients were divided into three primary groups for further analysis: those receiving CT alone, CRT, and surgical‐based treatment (with or without CT and/or RT).

In accordance with the variables in NCDB files, information collected on each patient broadly included demographic, clinical, and treatment data. All statistical tests were two‐sided, with a threshold of *P* < 0.05 for statistical significance, and were performed using SAS (version 9.4, Cary, NC). Univariable and multivariable (stepwise) logistic regression modeling was utilized to determine characteristics that were predictive for receipt of surgical‐based therapy. The Kaplan‐Meier method was used for survival analysis, and comparisons between groups were performed with the log‐rank test. Overall survival (OS) was defined as the interval between the date of diagnosis and the date of death, or censored at last contact. Cox univariate and multivariate (stepwise) analyses were performed to determine factors associated with overall survival.

## 3 RESULTS

**Figure 1:**
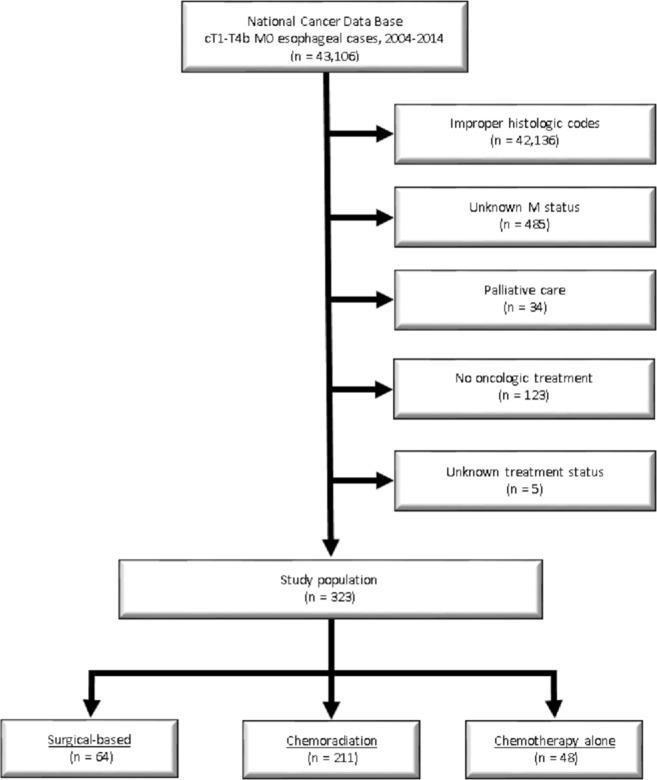
Patient selection diagram

A complete flow diagram of patient selection is provided in Figure 1. In total, 323 patients with nonmetastatic, pathologically‐proven ESCC met study criteria (Table 1). Of these, 64 (20%) patients underwent surgical‐based treatment, 211 (65%) CRT, and 48 (15%) CT alone.

**Table 1:** Characteristics of the overall cohort and factors associated with receiving surgery

Parameter	Surgery (N = 64)	CRT (N = 211)	CT Alone (N = 48)	Univariable	
				OR (95% CI)	*P*‐value
Age (years)					
Median (range)	63 (25‐86)	67 (35‐90)	68 (48‐87)	0.981 (0.959‐1.004)	0.101
Gender					
Male	44 (69%)	139 (66%)	29 (60%)	REF	‐
Female	20 (31%)	72 (34%)	19 (40%)	0.839 (0.467‐1.509)	0.558
Race					
White	57 (89%)	175 (83%)	40 (86%)	REF	‐
Black	7 (11%)	27 (13%)	4 (8%)	0.617 (0.264‐1.444)	0.266
Other	0 (0%)	9 (4%)	3 (6%)		
Charlson Deyo score^a^					
0	52 (81%)	151 (72%)	40 (83%)	REF	‐
1	8 (13%)	43 (20%)	6 (13%)	0.600 (0.267‐1.345)	0.422
≥2	4 (6%)	17 (8%)	2 (4%)	0.773 (0.252‐2.372)	0.998
Insurance type					
Uninsured	3 (5%)	4 (2%)	3 (6%)	REF	‐
Private	28 (44%)	69 (33%)	11 (23%)	0.817 (0.198‐3.376)	0.084
Medicaid/Other Government	1 (2%)	23 (11%)	2 (4%)	0.093 (0.008‐1.043)	**0.048**
Medicare	31 (48%)	111 (53%)	31 (65%)	0.509 (0.125‐2.081)	0.691
Unknown	1 (2%)	4 (2%)	1 (2%)	‐	‐
Income (US dollars/year)					
<$30 000	10 (16%)	46 (22%)	4 (8%)	REF	‐
$30 000‐$34 999	18 (28%)	49 (23%)	15 (31%)	1.406 (0.597‐3.313)	0.588
$35 000‐$45 999	18 (28%)	54 (26%)	18 (38%)	1.250 (0.533‐2.933)	0.962
≥$46 000	17 (27%)	56 (27%)	8 (17%)	1.328 (0.560‐3.152)	0.766
Unknown	1 (2%)	6 (3%)	3 (6%)	‐	‐
Location					
Metro	51 (80%)	162 (77%)	31 (65%)	REF	‐
Urban	9 (14%)	38 (18%)	12 (25%)	0.681 (0.314‐1.477)	0.057
Rural	3 (5%)	3 (1%)	0 (0%)	3.784 (0.724‐19.311)	0.070
Unknown	1 (2%)	8 (4%)	5 (10%)	‐	‐
Percentage of adults in zip code without high school diploma					
≥21%	11 (17%)	44 (21%)	3 (6%)	REF	‐
13‐20.9%	18 (28%)	48 (23%)	19 (40%)	1.148 (0.497‐2.653)	0.687
7‐12.9%	24 (38%)	71 (34%)	16 (33%)	1.179 (0.531‐2.615)	0.578
<7%	10 (16%)	42 (20%)	7 (15%)	0.872 (0.339‐2.244)	0.535
Unknown	1 (2%)	6 (3%)	3 (6%)	‐	‐
Facility type					
Community	29 (45%)	117 (55%)	27 (56%)	REF	‐
Academic	34 (53%)	89 (42%)	21 (44%)	1.535 (0.882‐2.671)	0.130
Unknown	1 (2%)	5 (2%)	0 (0%)	‐	**‐**
Facility location					
Northeast	13 (20%)	55 (26%)	10 (21%)	REF	‐
South	22 (34%)	62 (29%)	17 (35%)	1.392 (0.651‐2.978)	0.584
Midwest	17 (27%)	52 (25%)	17 (35%)	1.232 (0.555‐2.735)	0.999
West	11 (17%)	37 (18%)	4 (8%)	1.341 (0.549‐3.276)	0.761
Unknown	1 (2%)	5 (2%)	0 (0%)	‐	‐
Distance to treating facility (mi)					
Median (range)	17 (1‐1028)	9 (0‐1018)	8 (0‐66)	1.001 (0.999‐1.003)	0.346
Clinical T classification					
1	12 (19%)	25 (12%)	12 (25%)	REF	‐
2	6 (9%)	26 (12%)	2 (4%)	0.661 (0.221‐1.977)	0.739
3	23 (36%)	77 (36%)	4 (8%)	0.606 (0.275‐1.335)	0.416
4	0 (0%)	29 (14%)	7 (15%)		
Unknown	23 (36%)	54 (26%)	23 (48%)	‐	‐
Clinical N classification					
0	25 (39%)	67 (32%)	13 (27%)	REF	‐
1	19 (30%)	103 (49%)	10 (21%)	0.538 (0.278‐1.043)	0.343
2	3 (5%)	7 (3%)	3 (6%)	0.640 (0.171‐2.392)	0.835
3	0 (0%)	5 (2%)	0 (0%)		
Unknown	17 (27%)	29 (13%)	22 (46%)	‐	‐
Year of diagnosis					
2004‐2008	40 (63%)	99 (47%)	28 (58%)	REF	‐
2009‐2014	24 (38%)	112 (53%)	20 (42%)	0.577 (0.329‐1.012)	0.055

Statistically significant *P*‐values are in bold. Only values included in the final multivariable model are shown. CI, confidence interval; CRT, chemoradiotherapy; CT, chemotherapy; OR, odds ratio.

a The Charlson‐Deyo index is a weighted score of comorbidities as defined by several medical codes.

Due to the controversial role of surgery,^29^ univariable logistic regression analysis was performed to evaluate factors associated with receiving surgery. Patients with Medicaid/other (non‐Medicare) governmental insurance were less likely to undergo surgery (*P* = 0.048). However, patients with private insurance trended towards receipt of surgery (*P* = 0.084), along with those living in rural (*P* = 0.070) and nonurban (*P* = 0.057) areas. Treatment at more recent time periods also trended toward decreased use of surgery (*P* = 0.055). Likely related to sample size issues, no single factor significantly predicted for administration of surgery on multivariable analysis.

**Figure 2:**
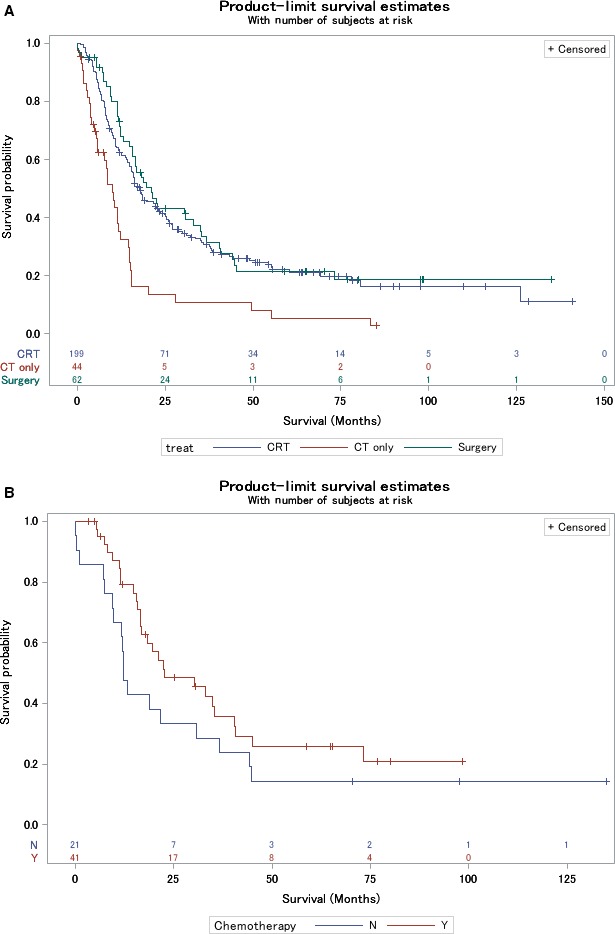
Kaplan‐Meier overall survival curves comparing surgery‐based treatment, chemoradiotherapy, and chemotherapy alone (A). Kaplan‐Meier overall survival curves of the surgery cohort stratified for delivery of additional chemotherapy (B)

Median follow‐up was 48 months (range, 1‐141 months). As shown in Figure 2A, there were no OS differences between the surgery‐based and CRT arms, but both were superior to CT alone (*P* < 0.001). The median OS for the surgery‐based cohort was 21 months (95% CI, 16‐33 months), as compared to 18 months (95% CI, 15‐23 months) for CRT, and 10 months (95% CI, 6‐12 months) for CT alone.

As part of additional subgroup analysis to investigate the potential impact of adjuvant CT after surgery, the 64 patients in the surgery cohort were subdivided into 43 patients that received CT and 21 that did not (39 underwent RT in any capacity, and 36 were given both CT and RT). Despite these small sample sizes, the impact of additional chemotherapy in the surgical cohort was assessed, with a nonsignificant difference between groups (Figure 2B; *P* = 0.143).

Owing to the similar survival between both groups involving local therapy (surgical‐based and CRT), these groups were combined for Cox multivariate analysis. Receipt of any local therapy independently predicted for higher OS (*P* < 0.001). Other factors independently associated with poorer OS included advancing age, increasing T and N classification, treatment at a community facility, and residence in an area with lower educational status (*P* < 0.05 for all) (Table 2).

**Table 2:** Univariate and multivariate Cox proportional hazards model for overall survival

Parameter (comparator vs reference)	Univariate			Multivariate		
	HR	95% CI	*P*‐value	HR	95% CI	*P*‐value
Treatment group (CT alone vs surgery/CRT)	2.276	1.602‐3.235	**<0.001**	2.824	1.711‐4.661	**<0.001**
Age (continuous)	1.013	1.002‐1.025	**0.025**	1.019	1.005‐1.034	**0.028**
Gender (male vs female)	0.928	0.706‐1.220	0.592			
Race (others vs white)	0.993	0.690‐1.430	0.971			
Charlson‐Deyo score (0 vs 2)	0.582	0.362‐0.937	**0.026**			
Charlson‐Deyo score (1 vs 2)	0.648	0.370‐1.134	0.129			
Distance to treating facility (continuous)	0.999	0.998‐1.001	0.390			
Insurance (private vs uninsured)	0.806	0.403‐1.612	0.542			
Insurance (Medicaid/other government vs uninsured)	1.336	0.608‐2.936	0.471			
Insurance (Medicare vs uninsured)	1.069	0.543‐2.105	0.847			
Income ($30 000‐$34 999 vs <$30 000)	1.312	0.883‐1.949	0.179			
Income ($35 000‐$45 999 vs <$30 000)	0.966	0.649‐1.438	0.864			
Income (≥$46 000 vs <$30 000)	0.978	0.652‐1.465	0.912			
Location (urban vs metro)	1.108	0.783‐1.569	0.563			
Location (rural vs metro)	1.031	0.423‐2.514	0.947			
Percentage of adults in zip code without high school diploma (13‐20.9% vs ≥21%)	0.788	0.512‐1.211	0.277	0.881	0.535‐1.450	0.618
Percentage of adults in zip code without high school diploma (7‐12.9% vs ≥21%)	0.968	0.658‐1.424	0.868	0.735	0.464‐1.165	0.190
Percentage of adults in zip code without high school diploma (<7% vs ≥21%)	0.715	0.494‐1.036	**0.076**	0.530	0.342‐0.823	**0.005**
Facility type (academic vs community)	0.698	0.537‐0.907	**0.007**	0.525	0.382‐0.721	**<0.001**
Facility location (South vs Northeast)	1.008	0.715‐1.421	0.964			
Facility location (Midwest vs Northeast)	1.034	0.720‐1.486	0.855			
Facility location (West vs Northeast)	0.894	0.587‐1.363	0.603			
T stage (T2 vs T1)	0.848	0.498‐1.444	0.544			
T stage (T3/4 vs T1)	1.135	0.773‐1.667	0.518			
N stage (N1 vs N0)	1.392	1.020‐1.898	**0.037**	1.568	1.134‐2.167	**0.007**
N stage (N2/3 vs N0)	2.620	1.403‐4.894	**0.003**	3.834	1.982‐7.418	**<0.001**
Year of diagnosis (2009‐2014 vs 2004‐2008)	0.945	0.725‐1.232	0.678			

Statistically significant *P* values are in bold. Only values included in the final multivariate model are shown. CI, confidence interval; HR, hazard ratio.

## 4 DISCUSSION

The study of rare neoplasms such as ESCC is highly limited by sample size and heterogeneity in existing reports; it is hence essential to perform large‐volume investigations of homogeneous patients. Our study of a contemporary national database, the largest study to date, demonstrates that most nonmetastatic ESCC is treated with CRT in the United States. As compared to CT alone, delivery of additional local therapy in the form of RT or surgery is associated with improved survival. The findings of this study corroborate elements from smaller studies.^6^, ^7^

It is important to mention that patients coded as undergoing palliative treatment were excluded from this study, so it is less likely that the CT alone group experienced poor survival from receiving palliative CT. The improved OS with the addition of RT to CT (also observed by Song et al.^5^) indicates that ESCC should be treated similar to limited‐stage small cell lung cancer, in which numerous prospective investigations have shown an OS benefit to adding RT to CT.^30^ However, this investigation also describes that treatment with an esophageal cancer paradigm may also be appropriate, in the sense that surgical management (with or without CT) is also associated with improved OS over CT alone. By this token, owing to the numerically higher OS with surgery/CT over surgery alone (Figure 2B), it may be logically stated that surgery/CT would also have superior OS as compared to CT alone. Although efforts were made to evaluate whether surgery/CT was superior to surgery alone (Figure 2B), sample size issues likely contributed to a statistically insignificant comparison. It is thus also logical that similar OS would exist between surgery/CT (n = 43) and CRT (n = 211). The reason that these conservative interpretations were posited (instead of formal analyses) is that continually constructing more subgroups split sample sizes greatly, and thus do not make for statistically adequate direct comparisons.

When delivered with systemic therapy, the issue of surgical‐based treatment vs definitive RT is also important to address. This question has been addressed with randomized trials in esophageal squamous cell and adenocarcinomas, displaying improvements in local control but no differences in OS between groups.^31^, ^32^ The results of those studies may be less applicable to the modern era for several reasons, however, including use of old surgical and RT techniques, split‐course RT paradigms, and treatment based on responders to chemotherapy. Nevertheless, when applied to NCDB data, which do not carry information on local control, operative complications, and stent placement, the comparative value of either modality remains inconclusive. Although it is possible that CRT patients were not “fit” enough to undergo surgery, it is also possible that surgery was delivered to bulkier and “higher‐risk” disease (in spite of the many unknown values in T/N classification). Taken together, we recommend that the choice of local therapy be tailored individually, including use of multidisciplinary discussion and patient input.

The sample sizes in this investigation were still relatively small, and this is likely the cause of the inconclusive findings on multivariable logistic regression analysis. However, this relative balance between groups also indicated that propensity matching was not statistically prudent, which would further decrease sample sizes. Likewise, this study also cannot assess the utility of surgery/CT or surgery/RT vs surgery/CRT, because few patients received the former.

The independent association between treatment at an academic center and higher OS as found on Cox multivariate analysis has far‐reaching implications on patient counseling and management by both oncologists and referring providers. There are many potential reasons for this, not limited to greater multimodality coordination, streamlined and thorough diagnostic processes and multidisciplinary discussion, technical expertise, ancillary staff for closer clinical monitoring, and potentially the availability of salvage treatments (or clinical trials). Nevertheless, these findings could warrant revisions in patterns of patient education, and it is recommended that patients with rare tumors such as ESCC should be treated at academic institutions.

Although the NCDB provides a unique platform with which to study this rare disease, this investigation is not without additional shortcomings to those discussed above. First, this study is not powered to address optimal sequencing of systemic and local therapies, which would result in splitting the cohort's subgroups into even smaller sample sizes and highly inaccurate OS comparisons. To this end, performing a meta‐analysis of available studies, such as those discussed previously,^4^, ^5^, ^6^, ^7^ would be useful; however, the marked heterogeneity in the available literature (eg merging metastatic and nonmetastatic patients, accounting for chemotherapy, etc.) is a major roadblock to doing so. It is also a major source of bias insofar as patients receiving local therapy may have been carefully selected based on response to induction chemotherapy, which cannot be quantified in the NCDB. Second, the NCDB does not keep track of several other factors, including chemotherapy cycles/agents, performance/functional status, toxicities, postoperative complications, toxicity‐related deaths, or RT field design/volumes/techniques. No information is also provided regarding whether proper workup was performed in each case to rule out a lung primary (eg PET‐CT). Third, it is additionally of particular concern whether the tumor was a mixed small cell tumor or a “pure” small cell neoplasm, although published studies addressing this question in lung cancer differ on the prognostic/predictive impact.^33^, ^34^ Fourth, the NCDB does not allow for an assessment of subsequent lines of treatment (eg re‐irradiation, further systemic and/or targeted therapy), which could impact OS. Lastly, the NCDB also does not provide genomic information, which has proven to be of great utility in other esophageal neoplasms.^35^, ^36^ Nevertheless, the known shortcomings of a national large‐volume database, the first of its kind to date, do not diminish the necessity for further investigation.

## 5 CONCLUSIONS

This is the largest study to date evaluating patterns of care and outcomes of ESCC. In the United States, most nonmetastatic ESCC is treated with CRT. As compared to CT alone, delivery of additional local therapy in the form of RT or surgery is associated with improved survival.

## CONFLICT OF INTEREST

This has never been presented/published before in any form. All authors declare no conflicts of interest.
